# Commentary: Anatomical constitution of sense organs as a marker of mental disorders

**DOI:** 10.3389/fnbeh.2016.00056

**Published:** 2016-03-24

**Authors:** Thomas Schwitzer, Raymund Schwan, Florent Bernardin, Coline Jeantet, Karine Angioi-Duprez, Vincent Laprevote

**Affiliations:** ^1^Pôle Hospitalo-Universitaire de Psychiatrie, Centre Psychothérapique de NancyLaxou, France; ^2^EA7298, INGRES, Université de LorraineNancy, France; ^3^Institut National de la Santé et de la Recherche Médicale U1114, Fédération de Médecine Translationnelle de Strasbourg, Département de Psychiatrie, Centre Hospitalier Régional Universitaire de StrasbourgStrasbourg, France; ^4^Maison des Addictions, CHU NancyNancy, France; ^5^EA4432, InterPsy, Université de LorraineNancy, France; ^6^Service d'Ophtalmologie, CHU NancyNancy, France

**Keywords:** schizophrenia, retina, pattern electroretinogram, pathophysiology, dopamine

Research in neuroscience and psychiatry is limited by the difficulty to accurately access brain functioning. There is currently a need to develop new methods assessing the neurobiological underpinning of brain dysfunctions (London et al., 2013; Lavoie et al., 2014; Güell and Bernácer, 2015; Laprevote et al., 2015; Schwitzer et al., 2015a,b). On this basis, Güell and Bernácer in an exciting article recently discussed the relevance of studying visual perception in mental disorders and especially in schizophrenia (Güell and Bernácer, 2015). Among other deficits, they outlined retinal functional and anatomical deficits detected in schizophrenia using respectively flash electroretinogram (fERG) and optical coherence tomography (OCT). However, we would like to suggest herein that the pattern electroretinogram (PERG), a retinal functional recording, might provide a relevant complementary measurement to enhance understanding of biological mechanisms underlying brain disorders in schizophrenia.

Flash and pattern ERG allow for the assessment of specific cell types of the neural retina and give different information on the pathophysiology. Using a light stimulation, the fERG mainly assesses the electric biopotential evoked by the first stages of visual processing namely photoreceptors and bipolar-Müller cell complex (Holder et al., 2010). As previously found, these first stages are altered in schizophrenia (Warner et al., 1999; Balogh et al., 2008; Hébert et al., 2015). However, the fERG does not significantly provide information concerning the ganglion cells, the axons of which form the optic nerve. The ganglion cells constitute the ultimate retinal relay before the transmission of the visual information from the retina to the visual cortex. The functional properties of these cells can be assessed by the PERG using the central presentation of reversing black and white checkerboards (Bach et al., 2013). The On-Off organization of the ganglion cells receptive fields makes these cells particularly sensitive to the alternate changes in contrast levels of the checkerboards, and leads to large responses in the PERG (Holder et al., 2010). The electrical signal transmitted to ganglion cells originates from photoreceptor and bipolar cells and is under the influence of interneurons cells (amacrine and horizontal cells). The signal elicited at the ganglion cell level thus results from the integration of several retinal stages. Since it is more integrated there than at the photoreceptor and bipolar cell level we suggest that its measurement might be a useful complementary test to approach the neural function in schizophrenia. Furthermore, the layer of the ganglion cells is the first retinal stage providing information in the form of action potentials.

Trustworthy evidence underlines that PERG protocols are good candidates to investigate dopamine transmission, a neurotransmitter known to be involved in schizophrenia. Indeed, the manipulation of the contrast level of the reversing black and white checkerboards during PERG informs on retinal contrast processing, which is largely influenced by retinal dopaminergic amacrine cells (Djamgoz et al., 1997). Moreover, alterations of retinal contrast processing detected with reversal PERG stimulation were found in Parkinson's disease, a dopaminergic pathology (Garcia-Martin et al., 2014). Importantly, these changes appear to be good biological markers, inasmuch as they predict quality of life and disease severity in Parkinson's disease.

PERG shares several advantages with fERG. Measurements of PERG are non-invasive, relatively fast, easy-to-use, and inexpensive. Importantly, PERG also has some additional advantages relative to fERG. Unlike most protocols of fERG, there is no need to dilate the pupil, making the PERG less invasive than fERG, with no alteration of visual perception. Second, whereas the fERG is evaluated after adaptation periods in darkness and light, the PERG is entirely measured in light conditions without any adaptation period (Bach et al., 2013; McCulloch et al., 2015). These methodological advantages may facilitate the use of PERG in patients with schizophrenia. As the PERG contrast processing appears sensitive to dopaminergic dysfunction, it might represent a suitable approach for monitoring anti-psychotic response, an important part of patient care. Finally, the PERG can be coupled with other retinal measurements such as the fERG to provide a more thorough picture of the pathophysiology of the disease.

As previously described, measures of PERG assess the functional properties of the retinal ganglion cells, the axons of which form the optic nerve. Anatomical constitution and organization of these cells can be evaluated by retinal imaging techniques, namely OCT. Several studies found retinal nerve fiber layer (RNFL) thinning in schizophrenia, which was observed with spectral domain OCT (Chu et al., 2012; Lee et al., 2013; Silverstein et al., 2015). RNFL is constituted by the fibers of the optic nerve and its thinning is a direct reflection of a loss of ganglion cell axons. We suppose that anatomical alterations of retinal ganglion cells observed in schizophrenia could lead to functional deficits of these cells. Such impairments could be detected by PERG. Importantly, adding functional measurements like PERG to imaging techniques such as OCT may provide a potential structure-function correlation.

Although PERG might appear as a relevant measure to indirectly approach the pathophysiology of schizophrenia, there are several limitations and a large number of steps required to demonstrate the usefulness of this exam in the better understanding of the disease. To this date, PERG measurements have not been evaluated in schizophrenia patients yet and consequently there is no certainty that differences between patients and healthy controls would be observed. Accordingly, case-control studies with a large number of subjects and standardized protocols are needed to eventually demonstrate differences between patients and controls. This constitutes the starting point to investigate the relevance of this method. Additionally, the pathophysiology of schizophrenia remains to this date elusive and involves more complex mechanisms than only dopaminergic dysfunctions (Khandaker et al., 2015). As a consequence, only one exam such as PERG does not alone give sufficient information on central dysfunctions but should be coupled with other assessments to provide a clearer picture of the disease. Then, in the case where differences between groups would be observed, control groups of patients with other dopaminergic pathologies are crucial to conclude on the specificity of PERG. Also, as we can consider that it is impossible to have medication-free schizophrenia patients, the effects of different types of psychotropic drugs such as antipsychotics must be tested on PERG recordings to differentiate the potential effects of medications or disease. Finally, although there is currently an increasing interest for PERG assessments in psychiatric research (Schwitzer et al., 2015a, 2016), the precise mechanisms underlying PERG anomalies should be investigated to understand the biological underpinning of brain dysfunctions.

Approximately 0.5% of the population suffers from schizophrenia, which has psychiatric and cognitive consequences. A current challenge in neuroscience research is to develop reproducible measures providing an indirect access to the brain functioning. In the case of schizophrenia, there is an urgent need for biological markers enabling early detection of the disease. Eventually, the PERG might be used as a useful complementary measurement to allow a better understanding of the pathophysiology of schizophrenia.

## Author contributions

All authors listed, have made substantial, direct, and intellectual contribution to the work, and approved it for publication.

### Conflict of interest statement

The authors declare that the research was conducted in the absence of any commercial or financial relationships that could be construed as a potential conflict of interest.

## References

[B1] Bach M. Brigell M. G. Hawlina M. Holder G. E. Johnson M. A. McCulloch D. L.. (2013). ISCEV standard for clinical pattern electroretinography (PERG): 2012 update. Doc. Ophthalmol. Adv. Ophthalmol. 126, 1–7. 10.1007/s10633-012-9353-y 23073702

[B2] Balogh Z. Benedek G. Kéri S. (2008). Retinal dysfunctions in schizophrenia. Prog. Neuropsychopharmacol. Biol. Psychiatry 32, 297–300. 10.1016/j.pnpbp.2007.08.024 17889979

[B3] Chu E. M.-Y. Kolappan M. Barnes T. R. E. Joyce E. M. Ron M. A. (2012). A window into the brain: an *in vivo* study of the retina in schizophrenia using optical coherence tomography. Psychiatry Res. 203, 89–94. 10.1016/j.pscychresns.2011.08.011 22917503 PMC4024658

[B4] Djamgoz M. B. Hankins M. W. Hirano J. Archer S. N. (1997). Neurobiology of retinal dopamine in relation to degenerative states of the tissue. Vision Res. 37, 3509–3529. 10.1016/S0042-6989(97)00129-6 9425527

[B5] Garcia-Martin E. Rodriguez-Mena D. Satue M. Almarcegui C. Dolz I. Alarcia R.. (2014). Electrophysiology and optical coherence tomography to evaluate Parkinson disease severity. Invest. Ophthalmol. Vis. Sci. 55, 696–705. 10.1167/iovs.13-13062 24425856

[B6] Güell F. Bernácer J. (2015). Anatomical constitution of sense organs as a marker of mental disorders. Front. Behav. Neurosci. 9:59. 10.3389/fnbeh.2015.00059 25805979 PMC4354419

[B7] Hébert M. Mérette C. Paccalet T. Émond C. Gagné A.-M. Sasseville A.. (2015). Light evoked potentials measured by electroretinogram may tap into the neurodevelopmental roots of schizophrenia. Schizophr. Res. 162, 294–295. 10.1016/j.schres.2014.12.030 25579051

[B8] Holder G. E. Celesia G. G. Miyake Y. Tobimatsu S. Weleber R. G. International Federation of Clinical Neurophysiology (2010). International Federation of Clinical Neurophysiology: recommendations for visual system testing. Clin. Neurophysiol. Off. J. Int. Fed. Clin. Neurophysiol. 121, 1393–1409. 10.1016/j.clinph.2010.04.010 20558103

[B9] Khandaker G. M. Cousins L. Deakin J. Lennox B. R. Yolken R. Jones P. B. (2015). Inflammation and immunity in schizophrenia: implications for pathophysiology and treatment. Lancet Psychiatry 2, 258–270. 10.1016/S2215-0366(14)00122-9 26359903 PMC4595998

[B10] Laprevote V. Schwitzer T. Giersch A. Schwan R. (2015). Flash electroretinogram and addictive disorders. Prog. Neuropsychopharmacol. Biol. Psychiatry 56:264. 10.1016/j.pnpbp.2014.04.005 24732440

[B11] Lavoie J. Maziade M. Hébert M. (2014). The brain through the retina: the flash electroretinogram as a tool to investigate psychiatric disorders. Prog. Neuropsychopharmacol. Biol. Psychiatry 48, 129–134. 10.1016/j.pnpbp.2013.09.020 24121062

[B12] Lee W. W. Tajunisah I. Sharmilla K. Peyman M. Subrayan V. (2013). Retinal nerve fiber layer structure abnormalities in schizophrenia and its relationship to disease state: evidence from optical coherence tomography. Invest. Ophthalmol. Vis. Sci. 54, 7785–7792. 10.1167/iovs.13-12534 24135757

[B13] London A. Benhar I. Schwartz M. (2013). The retina as a window to the brain-from eye research to CNS disorders. Nat. Rev. Neurol. 9, 44–53. 10.1038/nrneurol.2012.227 23165340

[B14] McCulloch D. L. Marmor M. F. Brigell M. G. Hamilton R. Holder G. E. Tzekov R.. (2015). ISCEV Standard for full-field clinical electroretinography (2015 update). Doc. Ophthalmol. 130, 1–12. 10.1007/s10633-014-9473-7 25502644

[B15] Schwitzer T. Lavoie J. Giersch A. Schwan R. Laprevote V. (2015a). The emerging field of retinal electrophysiological measurements in psychiatric research: a review of the findings and the perspectives in major depressive disorder. J. Psychiatr. Res. 70, 113–120. 10.1016/j.jpsychires.2015.09.003 26424430

[B16] Schwitzer T. Schwan R. Angioi-Duprez K. Giersch A. Laprevote V. (2016). The endocannabinoid system in the retina: from physiology to practical and therapeutic applications. Neural Plast. 2016:2916732. 10.1155/2016/2916732 26881099 PMC4736597

[B17] Schwitzer T. Schwan R. Angioi-Duprez K. Ingster-Moati I. Lalanne L. Giersch A.. (2015b). The cannabinoid system and visual processing: a review on experimental findings and clinical presumptions. Eur. Neuropsychopharmacol. J. Eur. Coll. Neuropsychopharmacol. 25, 100–112. 10.1016/j.euroneuro.2014.11.002 25482685

[B18] Silverstein S. Keane B. Rosen R. Paterno D. Metgud S. Cherneski L.. (2015). Relationships between indices of retinal thinning as revealed by spectral domain optical coherence tomography, and visual and cognitive impairments in schizophrenia. J. Vis. 15:590. 10.1167/15.12.59026326278

[B19] Warner R. Laugharne J. Peet M. Brown L. Rogers N. (1999). Retinal function as a marker for cell membrane omega-3 fatty acid depletion in schizophrenia: a pilot study. Biol. Psychiatry 45, 1138–1142. 10.1016/S0006-3223(98)00379-5 10331105

